# Disparities in Spinal Muscular Atrophy-Related Mortality in the United States, 2018–2023

**DOI:** 10.3390/neurosci7010022

**Published:** 2026-02-03

**Authors:** Ali Al-Salahat, Rohan Sharma

**Affiliations:** Neurology Department, Creighton University, Omaha, NE 68178, USA

**Keywords:** muscular atrophy, spinal, spinal muscular atrophies of childhood, mortality, health disparities, health inequities, neuromuscular diseases

## Abstract

Background: Prior SMA mortality studies have shown excess mortality in people with SMA, but the literature lacks data on disparities in SMA-related mortality. This study examined disparities in SMA-related mortality in the United States in the post-treatment era (2018–2023). Methods: This was a population-based study using the CDC Wide-ranging Online Data for Epidemiologic Research (CDC WONDER) database. The International Classification of Disease (ICD), 10th Revision, Clinical Modification codes, G12.0, G12.1, G12.8, and G12.9, were used to identify SMA. The data were stratified by biological sex, race/ethnicity (Non-Hispanic/NH White, NH Black, Hispanic, Asian) and Census regions (West, Northeast, Midwest, South). The analysis was conducted by calculating rate ratios (RR) of age-adjusted mortality rate (AAMR). Results: There were 821 (45.8% female) SMA-related deaths across the study period. Males were associated with higher AAMR than females (RR = 1.189, 95% CI: 1.035 to 1.366). The SMA-AAMR for NH White individuals was the highest compared to Hispanic individuals (RR = 1.808, 95% CI: 1.420 to 2.300), followed by NH Black and Asian individuals. The West carried the highest AAMR compared to the Northeast (RR = 1.581, 95% CI: 1.263 to 1.978), followed by the Midwest and the South. The age at death distribution showed a bimodal pattern, as follows: 5–14 years and 65–74 years. The infant age group (<1 year) was associated with the highest AAMR compared to all other age groups. Conclusion: Our findings showed that SMA-related mortality was highest in infants, NH White individuals, the West, and males. These data may assist future efforts to reduce the burden of SMA.

## 1. Introduction

Spinal muscular atrophy (SMA) is a progressive autosomal recessive neuromuscular condition that affects the alpha motor neurons in the anterior horns of the spinal cord [1]. It is caused by mutations or deletions in the SMN1 gene, leading to a deficiency in the survival motor neuron (SMN) protein [1,2]. Disease severity inversely correlates with SMN2 copy number [1,2]. SMA remains one of the most important genetic causes of infant mortality [2,3]. However, recent developments in the treatment and early diagnosis of SMA have led to a major shift in the survival of patients affected by SMA [1,2,3]. Individuals with SMA types 3 and 4 survive into adulthood, while those with SMA types 1 and 2 die in infancy and/or childhood [2]. The incidence of SMA is estimated at 1 in 6000–11,000 live births, worldwide [3]. The Global Burden of Disease study showed that the first year of life had the highest SMA-related mortality, consistent with type 1 SMA [4]. The clinical heterogeneity of SMA is determined by the SMN2 gene copy number, compensating for the loss of SMN1 function [1,2]. SMN1 produces a full-length and functional SMN protein, but SMN2 generates a truncated and unstable protein due to alternative splicing [1]. Approximately 10–15% of SMN2 transcripts do produce functional protein [2]. Therefore, individuals with higher SMN2 copy numbers have milder disease phenotypes. This genetic variation creates a spectrum of SMA severity, ranging from the most severe (type 0) to the mildest form (type 4), which mostly manifests during adulthood [1,2]. SMA type 1, or Werdnig–Hoffmann disease, accounts for 60% of all SMA cases. Infants with Werdnig–Hoffmann disease present within the first 6 months of life with hypotonia, weakness, and developmental motor delays [1,2,3]. The median survival is less than two years, usually from respiratory failure. SMA type 2 (Dubowitz disease) presents later, between 6 and 18 months of age, while SMA type 3 is usually manifested after 18 months of age [1,2]. Lastly, type 4 SMA usually presents during adulthood and is associated with a normal life expectancy [1,2,3].

Three FDA-approved disease-modifying therapies have been studied in SMA to increase SMN protein levels [5]. Along with newborn screening and early diagnosis, these treatments have transformed SMA into a treatable condition [5,6]. The three approved disease-modifying therapies for SMA include nusinersen (an anti-sense oligonucleotide), onasemnogene abeparvovec (gene replacement therapy), and risdiplam (an oral small molecule splicing modifier). Nusinersen was the first approved therapy in December 2016 [7,8,9,10]. It is administered via intrathecal injection with four loading doses followed by maintenance doses every 4 months. Onasemnogene abeparvovec also received FDA approval in May 2019, while risdiplam was approved in August 2020 [1,7,8,9,10]. The ENDEAR, STR1VE-US, and SUNFISH trials all showed the effectiveness of these three treatments in improving survival and lowering permanent ventilation requirements. Indeed, these treatments have transformed SMA from a lethal condition to one with therapeutic options [1,7,8,9,10].

The paradigm change from these disease-modifying therapies cannot be over-emphasized. The ENDEAR trial showed that infants with SMA type 1 treated with nusinersen had significantly improved survival compared to controls, with 41% of infants achieving motor milestones compared to 0% in the no-treatment arm [7,8,9,10]. The STR1VE trial of onasemnogene abeparvovec also showed that gene replacement therapy helped presymptomatic infants achieve developmental milestones comparable to normally developing children; 92% of children were sitting independently and 83% were walking independently at 18 months of age [1,7,8,9,10]. Lastly, the SUNFISH trial showed risdiplam’s efficacy as the first oral therapeutic option for SMA types 2 and 3 [1].

A previous epidemiological study on mortality from SMA in the pre-treatment era showed excess all-cause mortality in people with SMA [11]. However, the literature lacks epidemiological data on demographic disparities in SMA-related mortality in the United States (US). It is now time to evaluate these disparities in the post-treatment era. This study examined nationwide demographic disparities in SMA-related mortality based on biological sex, race/ethnicity, regions, and age groups from 2018 to 2023. This time period represents the post-treatment era in SMA.

## 2. Materials and Methods

This study followed the RECORD reporting guidelines. This was a retrospective population-based study that utilized data extracted from the CDC WONDER database. We extracted data on SMA-related deaths in the US from 2018 to 2023 using the multiple-causes-of-death files. The CDC WONDER multiple-cause-of-death files are divided into 1999–2020 and 2018–2023. We only extracted the 2018–2023 data, which includes the most recent data recorded after the initiation of the newer SMA treatments. The data is derived from death certificates filed in state vital statistics offices [12]. The denominator used for the mortality data is the entire US population and the Census Bureau estimates [13]. To identify SMA, we used the following International Classification of Disease (ICD), 10th Revision, Clinical Modification codes: G12.0, G12.1, G12.8, and G12.9 [14]. We obtained data regarding overall mortality, crude number of SMA-related deaths, crude mortality rate (CMR), and age-adjusted mortality rate (AAMR) per 1,000,000 population. The data were stratified by biological sex, race/ethnicity, US Census regions, and ten-year age groups. The AAMR controls for the variation in age distribution to allow data comparison; it was standardized using the 2000 US population. Race/ethnicity groups included Non-Hispanic (NH) White, NH Black, Asian, and Hispanic. Age groups were divided into ten-year groups, but the following groups were analyzed separately: <1 year, 1–4 years. Regions were divided into West, South, Northeast, and Midwest.

Analysis was conducted using mortality data of AAMR and CMR related to SMA, stratified by biological sex, race/ethnicity, regions, and age groups. For each group, we estimated rate ratios (RR) by dividing the rate in the group of interest by the rate in a pre-specified reference group. The following groups were considered as references: Hispanic, female, and Northeast. The CDC WONDER database provides 95% confidence intervals (CI) for each AAMR or CMR extracted, in addition to a standard error. This enabled 95% CI to be obtained for each RR calculated. Statistical significance was defined as *p* < 0.05. Age at death distribution was represented using grouped counts and visualized with a histogram-style bar chart. Figures, including bar charts and line plots of age-specific CMR with 95% CI, were generated using Python 3.11.6 (pandas and matplotlib).

## 3. Results

### 3.1. Overall Mortality and Sex-Based Disparities

**Figure 1:**
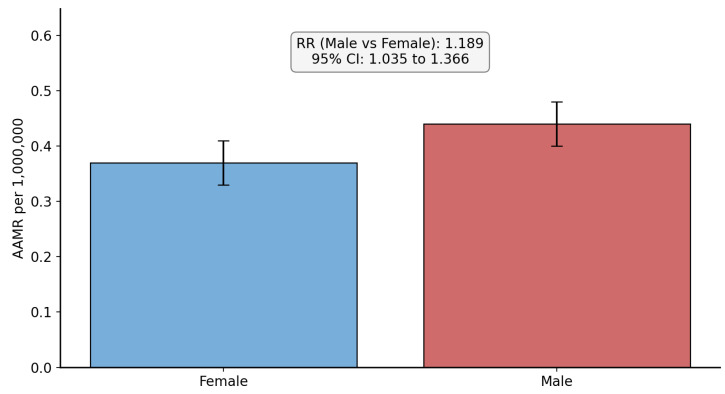
Bar chart comparing spinal muscular atrophy-related age-adjusted mortality rate (per 1,000,000 population) between female and male individuals. AAMR = age-adjusted mortality rate, RR = rate ratio, CI = confidence interval. Blue bar indicates the reference used for comparison.

Over the study period, there was a total of 821 SMA-related deaths in the US, with 565 deaths showing SMA as the underlying cause of death. Out of these deaths, 376 (45.8%) involved female individuals. The AAMRs for males and females were 0.44 and 0.37, respectively. When comparing male to female AAMRs, the RR was 1.189 (95% CI: 1.035 to 1.366, *p* = 0.014). Figure 1 shows the results of the male versus female comparison. Table 1 summarizes the SMA-related deaths and AAMR data with stratifications.

### 3.2. Race/Ethnicity-Based Disparities

**Figure 2:**
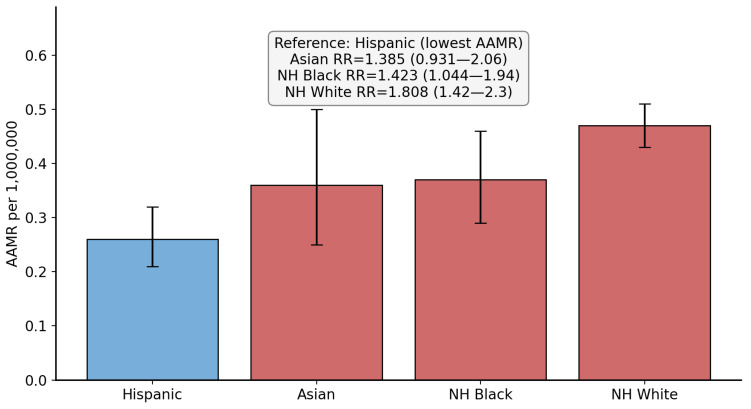
Bar chart showing the race/ethnicity-based comparison in spinal muscular atrophy-related age-adjusted mortality rate (per 1,000,000 population). AAMR = age-adjusted mortality rate, RR = rate ratio. Blue bar indicates the reference used for comparison.

The majority of the SMA-related deaths involved NH White individuals (79.8%). Hispanic individuals were associated with the lowest AAMR and were used as a reference group for comparison. Compared to Hispanic individuals, the RR for NH White, NH Black, and Asian individuals were 1.808 (95% CI: 1.420 to 2.300, *p* = 0.0000014), 1.423 (95% CI: 1.044 to 1.940, *p* = 0.026), and 1.385 (95% CI: 0.931 to 2.060, *p* = 0.108), respectively. Figure 2 demonstrates the race/ethnicity comparison. Stratification by other race/ethnic groups was not possible due to low and suppressed counts as per CDC WONDER guidelines.

### 3.3. Region-Based Disparities

**Figure 3:**
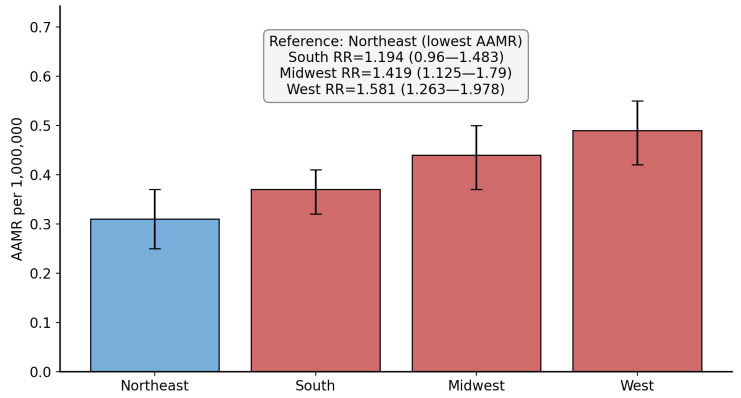
Bar chart showing the Census region-based comparison of spinal muscular atrophy-related age-adjusted mortality rate (per 1,000,000 population). AAMR = age-adjusted mortality rate, RR = rate ratio. Blue bar indicates the reference used for comparison.

The South was associated with the highest number of crude SMA-related deaths (289, 35.2%). The lowest AAMR was noted in the Northeast (0.31 per 1,000,000 population). Therefore, using the Northeast as reference, the RR for the West (RR = 1.581, 95% CI: 1.263 to 1.978, *p* = 0.00000639) was the highest, followed by the Midwest (RR = 1.419, 95% CI: 1.125 to 1.790, *p* = 0.003094) and the South (RR = 1.194, 95% CI: 0.96 to 1.483, *p* = 0.11045). Figure 3 shows the US Census region disparities in SMA-related mortality. Stratification by US States was not possible due to low and suppressed counts as per CDC WONDER guidelines.

### 3.4. Age-Group Based Comparison

**Figure 4:**
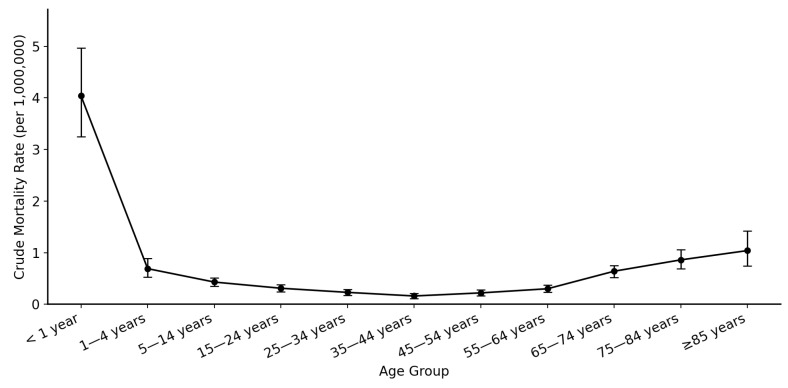
Plot chart showing the age group-based comparison of spinal muscular atrophy-related crude mortality rate (per 1,000,000 population). CMR = crude mortality rate; error bars represent 95% confidence interval.

**Figure 5:**
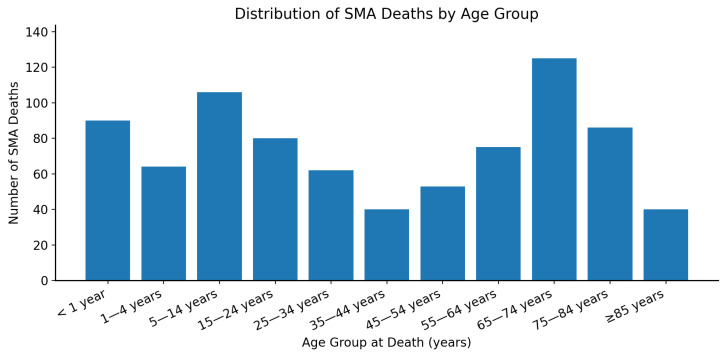
Bar chart showing the age at death distribution in spinal muscular atrophy-related deaths from 2018 to 2023 in the United States. SMA = spinal muscular atrophy.

A large proportion of SMA-related deaths occurred in the following age groups: 5–14 years (12.9%) and 65–74 years (15.2%). There were 90 deaths in infants (<1 year of age) and this group was associated with the highest CMR among all age groups (4.04 per 1,000,000 population), as shown in Figure 4. The distribution of age at death related to SMA showed an almost bimodal pattern, as shown in Figure 5. Table 2 summarizes SMA-related deaths and CMR stratified by age groups.

## 4. Discussion

This study revealed several important findings regarding SMA-related mortality in the US. We uncovered significant demographic disparities in SMA-related mortality in the post-treatment era. Notably, SMA-related mortality was found to be higher in male and NH White individuals, in the West, and in the infant age group. We also found a bimodal distribution of the age at death relating to SMA.

The literature is limited when it relates to sex-based differences in SMA prevalence and/or mortality. A Japanese study showed a larger number of male individuals with SMA type 3, while no difference was found for SMA type 1 or 2 [15]. Older studies from 1995 showed that males were affected by mild SMA more than females [16]. It is plausible that in the post-treatment era, male individuals with milder forms of SMA were less likely to be diagnosed early and receive disease-modifying treatment. This potential explains our findings of higher AAMR in males compared to females over the period 2018–2023. As a reinforcing example, an insurance claims study between 2008 and 2015 showed that adult patients with male infertility were diagnosed with mild SMA later in life [17]. Indeed, males affected by milder forms of SMA may remain undiagnosed through childhood and adolescence and are diagnosed later in life during infertility investigations. This delay in diagnosis may also delay possible interventions. Additionally, while SMA is an autosomal recessive condition and should affect both females and males equally, hormonal effects and sex-based modifiers that may potentially influence motor neuron health and SMN protein function remain unclear [15]. Future studies with granular-level patient data may establish sex-based differences in SMA clinical phenotypes and severity that explain our findings. Lastly, the sex-based differences we found were modest in magnitude, albeit statistically significant. Previous studies relating to SMA mortality were most likely underpowered to detect these differences.

Prevalence and carrier frequency studies have shown higher rates of SMA in European and Asian populations compared to African and Hispanic populations [18,19,20]. Our findings are consistent with these previous findings, as NH White individuals were associated with the highest AAMR in the race/ethnicity-based comparison. However, even though previous data show that NH Black individuals are among the groups with a lower prevalence and carrier frequency of SMA, we found that their associated SMA-AAMR was still higher than that of Hispanic individuals. This finding may point to possible care access-based disparities between different race/ethnicity SMA groups in the US. Data on the relation between race/ethnicity and severity/phenotype of SMA are lacking, but most of the available evidence suggests that the main determinants of severity are SMN2 copy number and supportive care access [21,22,23]. The disproportionate AAMR in NH Black individuals relative to their lower disease prevalence suggests systemic barriers to optimal care, including access to disease-modifying treatments and specialized multidisciplinary care. Moreover, the high cost of these treatments remains an important barrier in certain populations. Access to services, such as genetic counseling and carrier screening programs, is also problematic. Our findings underscore the importance of further investigating race/ethnicity differences in SMA phenotype, severity, and access to care and disease-modifying treatments. Nonetheless, it is most likely that the higher AAMR in NH White individuals reflects prevalence rather than true case fatality. The racial/ethnic disparities we identified in our study raise important questions about the multifactorial nature of health inequities in rare disease populations. Our findings suggest that structural and system-level factors contribute to disparities in SMA outcomes across different racial/ethnic groups.

Regarding regional-based differences in SMA, very limited data exist. Prevalence data has shown no regional differences among the US states [17]. However, there is substantial variation in the availability of SMA disease-modifying treatments across states, driven by Medicaid coverage policies. These variations in requirements for treatment eligibility are based on SMN2 copy number, ventilator status, and prescriber expertise [24]. This difference may impact patient access to treatment and, therefore, explain the significant region-based disparities in SMA-related mortality in our study. Even in states, such as New York, where statewide implementation of newborn screening facilitates early diagnosis and treatment, barriers related to insurance delays and infrastructure limitations persist [25]. Additionally, the rollout of these newborn screening programs was uneven across the US region, which led to disparities in early detection and treatment [26,27]. These aforementioned factors have probably played a role in the regional disparities we uncovered in our study. The higher mortality rate in the West is notable because this region generally has well-resourced medical centers, suggesting factors beyond healthcare infrastructure behind regional disparities. Furthermore, the administration of some of the disease-modifying treatments for SMA require specialized expertise. Nusinersen requires intrathecal administration by expert neurologists or interventional radiologists; these services can be difficult to access in certain regions. Onasemnogene abeparvovec also needs careful monitoring in specialized centers during administration. These resources are usually concentrated in large metropolitan areas, creating barriers for rural and underserved areas. These factors compound the regional disparities in SMA-related mortality. Our findings may assist healthcare administrators and policymakers in efforts related to equitable access to SMA specialized care and treatment. This study also highlights the lack of standardized national criteria for treatment access, which is a significant equity issue.

Future work from longitudinal cohort studies is needed to track mortality trends as the population of SMA patients ages, and the healthcare system develops improved access to novel therapies. Studies aimed at analyzing specific barriers to SMA specialized care in certain populations would help inform targeted interventions to improve equity. Upcoming work may also focus on sex-based drivers of mortality in SMA and differences in SMA phenotypes. Lastly, an aanalysis examining equity in resource allocation and cost of the SMA treatment along with insurance coverage may assist in mitigating disparities in SMA-related mortalities.

The limitations of this study included the reliance on ICD codes for identifying SMA, with potential misclassification bias. In addition, stratifying the data by the type of SMA was not possible given the nature of the ICD codes and the low overall counts in the study. Analyzing disparities in other racial and ethnic groups (NH Asian or Pacific Islander, NH American Indian or Alaska Native individuals, etc.) was limited because of suppressed data in each subgroup. The CDC suppresses counts of fewer than 10 in the CDC WONDER data to protect confidentiality, and mortality rates are marked unreliable for a count less than 20 per the CDC WONDER data use agreement. Moreover, caution is advised in the interpretation of the results as the analysis included in this study does not imply causal relationships and represent observed disparities. Lastly, the adult SMA population included in this study does not reflect post-treatment mortality differences as the use of the treatments is limited in adults.

## 5. Conclusions

This study represents the first population-based analysis of demographic disparities in SMA-related mortality in the post-treatment era in the US. The findings uncovered here may assist efforts in the equitable delivery and implementation of SMA specialized care and treatment and newborn screening programs. Policymakers and healthcare administrators may also find this data beneficial in improving regional disparities in SMA-related mortality. However, differences in prevalence may account for the observed disparities in mortality. Future studies may focus on granular-level data and identifying differences in SMA phenotypes based on biological sex and race/ethnicity and on the underlying/contributing causes of death in SMA in the post-treatment era.

## Figures and Tables

**Table 1:** Spinal muscular atrophy-related deaths and AAMR (2018–2023).

Characteristic	Deaths	AAMR per 1,000,000	RR (95% CI)	*p*-Value
Total	821	0.4	NA	NA
Biological Sex				
Female (Ref)	376	0.33	1	NA
Male	445	0.44	1.189 (1.035 to 1.366)	0.014
Race/Ethnicity				
Hispanic (Ref)	99	0.26	1	NA
NH White	574	0.47	1.808 (1.420 to 2.300)	<0.0001
NH Black	86	0.37	1.4230 (1.0438 to 1.9400)	0.0257
Asian	36	0.36	1.3846 (0.9306 to 2.0600)	0.1084
Census Region				
Northeast (Ref)	113	0.31	1	NA
South	289	0.37	1.1935 (0.9604 to 1.4831)	0.1104
Midwest	191	0.44	1.4193 (1.1254 to 1.7900)	0.003
West	228	0.49	1.5806 (1.2629 to 1.9783)	<0.0001

Abbreviations: NH = Non-Hispanic, RR = rate ratio, AAMR = age-adjusted mortality rate, Ref = reference, NA = not applicable, 95% CI = 95% confidence interval.

**Table 2:** Spinal muscular atrophy-related deaths and crude mortality rates by age group (2018–2023).

Age Groups	Deaths	CMR per 1,000,000	RR (95% CI)	*p*-Value
<1 year	90	4.04	25.25 (18.30 to 34.83)	<0.000001
1–4 years	64	0.69	4.31 (3.03 to 6.14)	<0.000001
5–14 years	106	0.43	2.69 (1.98 to 3.65)	<0.000001
15–24 years	80	0.31	1.94 (1.42 to 2.64)	<0.000001
25–34 years	62	0.23	1.44 (1.01 to 2.05)	0.045
35–44 years (Ref)	40	0.16	1	NA
45–54 years	53	0.22	1.38 (0.96 to 1.98)	0.085
55–64 years	75	0.3	1.88 (1.37 to 2.57)	<0.0001
65–74 years	125	0.64	4 (2.94 to 5.43)	<0.000001
75–84 years	86	0.86	5.38 (3.90 to 7.40)	<0.000001
85+ years	40	1.04	6.50 (4.41 to 9.59)	<0.000001
Total	821	0.41	NA	NA

Abbreviations: CMR = crude mortality rate, Ref = reference, NA = not applicable, 95% CI = 95% confidence interval.

## Data Availability

The original data presented in the study are publicly available upon request from the CDC WONDER database.
